# Personal

**Published:** 1890-03

**Authors:** 


### Personal.

Joe. Elmer Widner, of this city, was graduated in medicine on February 10, 1890, by Niagara University. He passed the regular examinations with credit at the close of last Winter's session, but could not be graduated with his class, because he was not twenty-one years old. Having attained his majority, the degree of Doctor of Medicine has been duly conferred, and we congratulate our young friend.

C. S. Ayres, A. M., M. D., the well-known oculist of Cincinnati, has been elected president of the Cincinnati Polyclinic Graduate School of Medicine. This institution could have no better assurance of success than calling such an able and widely known man to the presidency.

Dr. C. M. Daniels, who has been in Europe for the past six months, has returned, and will devote himself to the practice of surgery and gynecology.

Dr. Floyd S. Crego, of 280 Franklin street, is spending a few months in the Capitals of Europe, and is expected to return in May.

Dr. L. S. McMurtry has removed from Danville to Louisville, Ky. His office is at 652 Fourth avenue. Hours, 9—12.

Dr. G. W. York has removed to 190 Franklin street, Buffalo.

